# *m*RNA Expression of Two Colon Enzymes in Pre-Pubertal Gilts During a 42-Day Exposure to Zearalenone

**DOI:** 10.3390/toxins17070357

**Published:** 2025-07-17

**Authors:** Magdalena Gajęcka, Łukasz Zielonka, Maciej T. Gajęcki

**Affiliations:** Department of Veterinary Prevention and Feed Hygiene, Faculty of Veterinary Medicine, University of Warmia and Mazury in Olsztyn, Oczapowskiego 13, 10-718 Olsztyn, Poland

**Keywords:** zearalenone mycotoxicosis, *CYP1A1*, *GSTπ1*, ascending colon, descending colon, pre-pubertal gilts

## Abstract

The aim of this study was to determine whether a low dose of zearalenone (ZEN) affects the *m*RNA expression of the *CYP1A1* (P450 cytochrome) and *GSTπ1* (glutathione S-transferase) genes in the large intestine of pre-pubertal gilts. Materials: Control (C) group gilts (n = 18) received a placebo. Experimental (E) group gilts (n = 18) were orally administered 40 μg ZEN/kg body weight (BW) each day before morning feeding for 42 days. Three animals from each group were sacrificed each week of the study. Tissue samples were collected from the medial parts of the ascending colon and the descending colon on six dates. Results: Zearalenone concentrations were multiple times higher in the last three weeks of exposure, and ZEN metabolites were not detected. In phase I, *CYP1A1 m*RNA expression in the ascending colon was suppressed in the final three weeks of exposure, which substantially increased the ZEN concentration in the descending colon. In phase II, ZEN levels were high in the descending colon due to *CYP1A1* suppression in the ascending colon. Consequently, the phase II detoxification processes could not take place due to the absence of a substrate. Conclusion: This study demonstrated that low-dose ZEN mycotoxicosis disrupts the expression of the *CYP1A1* and *GSTπ1* genes, which co-participate in the enzymatic biotransformation of ZEN in both examined sections of the large intestine. The above could have contributed to increased ZEN accumulation in the mucosa of the descending colon in the last three weeks of exposure.

## 1. Introduction

Animal feeds are often a vector of mycotoxins (risk factors) that are transferred from feed raw materials to living organisms, leading to mycotoxin poisoning, namely subclinical mycotoxicosis. Mycotoxicosis is caused by the acute or prolonged exposure to one or more mycotoxins that are ingested at a given dose and over a certain period of time. Hundreds of mycotoxins have been identified to date, but only less than twenty mycotoxins present in plant materials give serious cause for concern due to their unpredictable implications for mammal health. This group of compounds includes undesirable substances produced by several dozen *Fusarium* species in cereal grain, including wheat and maize. Trichothecenes (including deoxynivalenol, T-2 toxin, nivalenol, verrucarin, and many others), fumonisins, patulin, and zearalenone (ZEN) are the most dangerous mycotoxins with adverse health effects [1].

Zearalenone is a toxic secondary metabolite of a large number of mold fungi of the Fusarium genus with the molecular formula C_18_H_22_O_5_. This white crystalline compound has a melting point of 161–163 °C and is characterized by low polarity. Zearalenone is soluble in fats and alkaline aqueous solutions, but almost insoluble in water. When ingested by animals, during the biotransformation processes of ZEN, many metabolites are formed, such as α- and β-zearalenol (α-ZEL and β-ZEL). These metabolites, also known as modified mycotoxins, have specific biological activity and exert significant toxicity [2,3]. Both the parent composition and its metabolites structurally resemble estrogen hormones. Zearalenone and its metabolites can bind to estrogen receptors (ER-α and ER-β) by competing for binding sites with 17-β-estradiol. Therefore, ZEN, α-ZEL, and β-ZEL possess estrogenic activity. Exposure to ZEN may lead to pathological changes in cells and tissues [4,5]. The pathogenic effects of ZEN include estrogenic, cytotoxic, and neurotoxic activity; it disrupts the intestinal barrier function, modulates the immune responses, and cooperates with the development of liver and colon cancer [1,6,7,8,9].

When living organisms are exposed to ZEN, various mechanisms are initiated in the body to remove or neutralize this undesirable compound. If systemic homeostasis is disrupted, ZEN can accumulate in the body and reach toxic levels. Macroorganisms initiate protective mechanisms by biotransforming mycotoxins into hydrophilic compounds that can be eliminated through the liver or kidneys [10]. This process is preceded by hyperestrogenism, namely hormone concentrations that exceed physiological levels, which contributes to a minor increase in total estradiol concentration, a decrease in the levels of other steroid hormones [8,11], or qualitative and quantitative changes in the microbiota colonizing the distal part of the colon [12,13,14,15,16].

Mycotoxins such as ZEN and its metabolites are transformed in cells by two classes of enzymes. The biotransformation process consists of two phases [17]. In phase I, cytochrome P450 (CYP) proteins add a reactive polar group to toxic compounds to transform them into more water-soluble forms. CYP enzymes are found throughout the body and are tissue-specific [18,19]. Genetic polymorphisms in CYP450 enzymes induce variations in their expression and activity [10]. At the same time, the metabolites produced in the presence of the CYP1A1 isoform activate transcription factors that induce (hypothetically) phase II enzymes such as GSTπ1 [10,20]. In phase I, the parent compound is eliminated from the body during the biotransformation process, but new toxic metabolites are formed [18] and bioactivated. According to the previous reports of Knutsen et al. [21], ZEN can act as both a substrate and an inhibitor of CYP. In turn, Kaci et al. [19] demonstrated that ZEN generally inhibits *CYP1A1 m*RNA expression.

In phase II of biotransformation, the efficiency of the processes is determined based on the activity of glutathione S-transferase (GST) [22]. Glutathione S-transferase belongs to the enzyme family that participates in the detoxification of different harmful compounds by catalyzing their conjugation with glutathione [23,24]. The maintenance of a balance in the processes regulated by GST plays a very important role in cellular homeostasis, and a dysregulation of these mechanisms can have significant implications for animal health [25]. The speed of biotransformation processes in which undesirable compounds are eliminated from the body influences the response of enterocytes to mycotoxins. GSTπ1 is a particular isoform of enzymes from the GST family. It may support glutathione (GSH) conjugation with unwanted compounds to detoxify these compounds, helping to maintain cellular homeostasis [23]. To produce reduced GSH conjugates, GSH must bind to reactive electrophiles obtained during cytochrome P450 metabolism. This process underscores the cytoprotective role of GST. The conjugated GSH compounds are then transported to the kidneys or excreted in the bile. They also participate in the metabolism of steroid hormones and the maintenance of GSH homeostasis [26].

In addition, the discussed enzymes have been identified in tumors, tumor cells, and tumor cell lines. Most antineoplastic drugs are metabolized by these enzymes [27]. It is not known whether ZEN alters the expression of the genes modulating enzyme activity in the large intestine, such as CYP1A1 and GSTπ1 [17]. Zearalenone and its metabolites activate metabolic processes by altering the expression of ER-β in the colon [28], thus potentially contributing to inflammation of the intestinal mucosa and, with chronic exposure, an increased risk of neoplastic conditions or provoking processes associated with neoplastic transformation. Therefore, the authors of this study hypothesized that ZEN mycotoxicosis can lead to the inhibition or activation of the discussed enzymes, thus contributing to neoplastic changes in the distal part of the colon.

To validate the above hypothesis, the aim of this in vivo study was to determine whether exposure to a ZEN dose of 40 µm/kg BW over a period of 42 days affects the expression of genes modulating the activity of CYP1A1 and GSTπ1 enzymes in the mucosa of selected segments of the large intestine (ascending colon and descending colon) in pre-pubertal gilts.

## 2. Results

### 2.1. Feed

The feed administered to both groups did not contain mycotoxins (ZEN or deoxynivalenol (DON) and its metabolites—(ZEN- and DON-free)) and mycotoxin strength was below the sensitivity of the method, i.e., the contamination of DON was below 2 ng/kg of feed, and the contamination of ZEN was below 5 ng/kg of feed. Masked mycotoxin concentrations were not analyzed.

### 2.2. Clinical Signs

No clinical signs of zearalenone mycotoxicosis were observed during the experiment. The histopathological, genetic, and ultrastructural examinations of tissue samples collected from the same animals revealed a number of changes. The results of these analyses were presented in anterior studies [22,29].

### 2.3. Strength of ZEN and Its Metabolites

**Figure 1:**
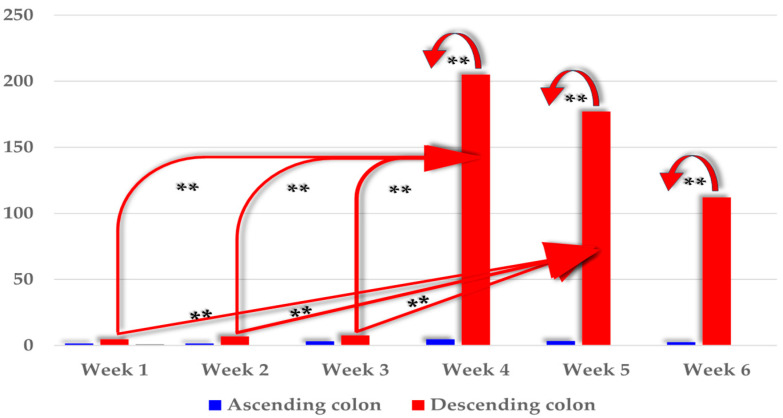
The concentrations of ZEN (ng/g) in the ascending and descending colon of pre-pubertal gilts during six weeks of exposure (1–6). Zearalenone levels are presented as mean (±) and standard deviation (SD) in each week. ZEN concentrations were compared between the tissues at specific exposure dates. Statistically significant differences were found: ** *p* ≤ 0.01.

Mycotoxin strength was analyzed in the distal colon of pre-pubertal gilts orally administered ZEN. Statistical differences were found in the strength of the parent compound (ZEN) between the blood sampling dates (Figure 1), but no ZEN metabolites (α-ZEL and β-ZEL) were found.

The differences between the mean ZEN levels in the examined tissue (ascending colon or descending colon) in each week of exposure in animals administered the same ZEN doses were analyzed first.

In the ascending colon, ZEN strength was very low during each week of exposure at 2.09 ng ZEN/g on average (Figure 1). No significant differences were found.

In the descending colon, the mean ZEN strength was very low in the first three weeks of exposure (Figure 1) at 6.4 ng ZEN/g on average. No statistical differences were found. In the last three weeks of exposure (weeks 4, 5, and 6), the ZEN concentrations increased sharply (with a decreasing trend) in the studied tissue (to 205.01 ng ZEN/g, 177.01 ng ZEN/g, and 112.01 ng ZEN/g, respectively). Significant differences (*p* ≤ 0.01) in ZEN concentration were noted between the first three weeks of exposure (weeks 1, 2, and 3) and week 4 (difference of 200.31 ng ZEN/g, 198.21 ng ZEN/g, and 197.44 ng ZEN/g, respectively) and week 5 (difference of 172.31 ng ZEN/g, 170.21 ng ZEN/g, and 173.61 ng ZEN/g, respectively).

At the same time, significant differences between mean ZEN concentrations between the examined sections of the large intestine were also analyzed in each week of exposure in animals receiving the same ZEN doses. The mean ZEN levels differed significantly (*p* ≤ 0.01) between the compared segments of the large intestine only in weeks 4, 5, and 6 (difference of 200.33 ng ZEN/g, 173.61 ng ZEN/g, and 109.55 ng ZEN/g, respectively).

### 2.4. Gene Expression of CYP1A1 and GSTπ1 Enzymes

**Figure 2:**
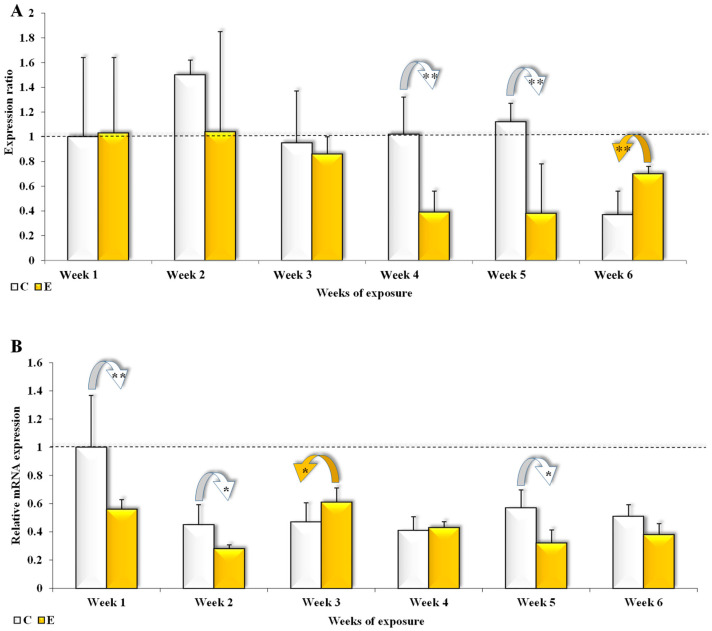
The expression of the *CYP1A1* gene in the colon (ascending colon (**A**) and descending colon (**B**)) in different weeks of the experiment (1–6). In each sample, enzyme expressions are presented as mean values (±) and standard deviation (SD) compared with the control sample at the beginning of the experiment (ER = 1.00; dashed line). Statistically significant differences: * *p* ≤ 0.05 and ** *p* ≤ 0.01.

CYP1A1 gene expression was suppressed in both analyzed parts of the colon (Figure 2) and in both groups throughout the experiment. The degree of suppression was greater in group E in both sections of the colon, in four out of the six weeks of the experiment. In the ascending colon (Figure 2A), significant (*p* ≤ 0.01) differences (0.98 and 1.16, respectively) were found in weeks 4 and 5. In week 6 (difference of 0.52), the observed difference (0.33) was also significant (*p* ≤ 0.01) in group E (Figure 2A).

In the descending colon (Figure 2B), the differences in the expression of the *CYP1A1* gene were significantly higher (*p* ≤ 0.05) in group C in weeks 2 and 5 (0.14 and 0.24, respectively) and in group E in week 3 (0.13). Significant differences (*p* ≤ 0.01) in group C were higher in week 1 (by 0.41) (Figure 2B).

In groups C and E, the mean CYP1A1 gene expression during the entire experiment was at 0.99 and 0.73 for the ascending colon and 0.57 and 0.43 for the descending colon, respectively.

**Figure 3:**
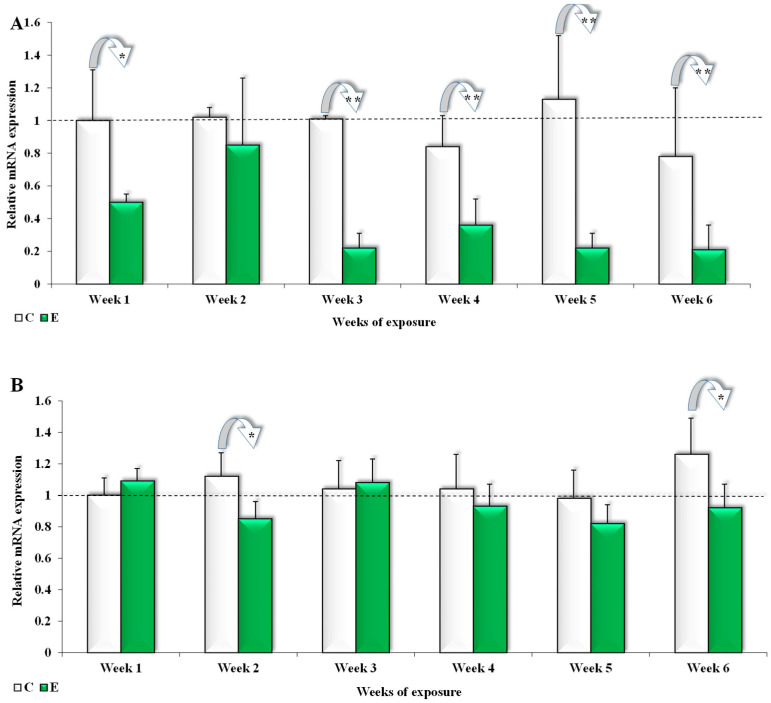
The expression of the *GSTπ1* gene in the colon (ascending colon (**A**) and descending colon (**B**)) in different weeks of the experiment (1–6). In each sample, enzyme expressions are presented as mean values (±) and standard deviation (SD) compared with the control sample at the beginning of the experiment (ER = 1.00; dashed line). Statistically significant differences: * *p* ≤ 0.05 and ** *p* ≤ 0.01.

In the ascending colon (Figure 3A), the differences in the expression of the *GSTπ1* gene in group C were significantly higher (*p* ≤ 0.05) in week 1 (0.35). In group E, the significant differences (*p* ≤ 0.01) were lower in weeks 1, 3, 4, and 5 (0.6, 0.97, 0.59, 1.1, and 0.7, respectively) (Figure 3A).

In the second and sixth week of exposure, significant differences (*p* ≤ 0.05) in expression were observed; the *GSTπ1* gene (Figure 3B) in the descending colon was lower in group E (0.27 and 0.34, respectively). In the other weeks of the experiment, the average values of the analyzed factors were higher in group C, but the observed differences were not statistically significant (Figure 3B).

In groups C and E, the average expression of the *GSTπ1* gene during the entire experiment was 0.96 and 0.39 for the ascending colon and 1.07 and 0.94 for the descending colon, respectively.

## 3. Discussion

The effect of low doses of ZEN on the mRNA expression of enzymes in the colon has not been studied, and most studies have been conducted in vitro. The present study was performed in vivo, which is why the results are more difficult to interpret.

### 3.1. Biotransformation of ZEN

The results of this study point to certain inconsistencies in the maintenance of homeostasis in response to prolonged low-dose zearalenone mycotoxicosis in the ascending colon and descending colon. Mycoestrogen levels remained fairly balanced in the first three weeks of exposure (Figure 1). At weeks 1–3, ZEN concentrations in the colonic samples were generally low (on average from 2.09 ng ZEN/g from the ascending colon to 6.4 ng ZEN/g from the descending colon). The above inhibited the development of germ cells [30,31]. Starting at week 4, the ZEN levels in samples from the descending colon increased significantly (*p* ≤ 0.01) by 172.31 ng ZEN/g (Figure 1), which could be attributed to (i) the negative effects of estrogen synthesis outside the gonads [11,32] due to the conversion of androgens [33,34]; (ii) the weakening of adaptive mechanisms [35]; (iii) the more effective utilization of dietary protein and energy [36,37]; (iv) the slowing down of biotransformation processes [38].

The absence of ZEN metabolites in phase I of the biotransformation process was surprising, and it could have been caused by the physiological deficiency of steroid hormones. Pre-pubertal gilts were probably able to compensate for this deficiency before the enzymatic biotransformation of ZEN leading to detoxification. This hypothesis is confirmed by the fact that ZEN concentration increased significantly (*p* ≤ 0.01) in the descending colon after the third week of exposure (Figure 1). However, it cannot be excluded that the levels of ZEN metabolites were below the limit of detection [39].

On the other hand, even minor (subclinical) feed contamination with ZEN can stimulate proliferative processes in pre-pubertal gilts [11]. It is a good indicator of weight gain in pre-pubertal animals, which have to achieve a certain body weight before sexual maturation [5]. This observation confirms that the gastrointestinal tract regulates somatic health [34]. In this way, the digestive system acts as a “second brain” [40] as it has many roles, in particular modulating the signals (stimuli) between the intestinal contents, intestinal tissues, and the central nervous system [41]. However, the role of selected colon enzymes during low-dose zearalenone mycotoxicosis remains unknown.

### 3.2. Gene Expression of CYP1A1 and GSTπ1 Enzymes in the Colon

The biotransformation of xenobiotics, including ZEN, can alter the chemical properties and biological activity of compounds, in particular phase I metabolites with increased functionality. Phase II metabolites are characterized by much lower or no biological activity. However, the type and scope of these reactions differ across compounds, depending on their concentrations during exposure [42], and are susceptible to individual variations [43]. The biotransformation of ZEN (phases I and II) leads not only to its elimination from the body; both the parent compound and the substances produced during phase I biotransformation are also inactivated. Various types of enzymes participate in these processes. Cytochrome P450 isoenzymes and, in intestinal tissues, enzymes of the CYP1A family are involved in phase I biotransformation. Microsomal GST enzymes participate in phase II biotransformation [44].

#### 3.2.1. CYP1A1 Gene in Phase I of the Biotransformation Process

Cytochrome P450 isoforms, in particular enzymes of the CYP1A1 family, are involved in phase I of the enzymatic biotransformation process in cell membranes. Epigenetic factors, including xenobiotics and undesirable substances [45] such as ZEN, increase the risk of uncontrolled proliferation [46]. They can act as ligands and signaling molecules which activate receptors (e.g., ERs are activated by ZEN) and transform them into transcription factors that activate the expression of target genes when the receptor binds to a specific DNA sequence. This sequence is often found in the promoter region of genes encoding the production of cytochrome P450 1A1 family enzymes which metabolize mycotoxins [45]. It remains unknown whether ZEN (similarly to estradiol) is a substrate for cytochrome P450 1A1 [38]. In the present study, low-dose ZEN mycotoxicosis induced specific changes in *CYP1A1 m*RNA expression in both parts of the colon (Figure 2A and Figure 3A). The following explanations are possible: (i) ZEN and its metabolites (independent variables) present in feed could compete (for example, with estradiol) as substrates for the analyzed enzyme; (ii) ZEN and its metabolite, α-ZEL, could be highly involved in biotransformation processes; (iii) both processes could occur simultaneously.

The levels of estradiol, which is the strongest estrogen, determine the balance between estrogens and estrogen-metabolizing enzymes [47,48]. Therefore, the minor silencing of expression in group C (silencing was significantly higher in group E in the ascending colon—Figure 2A) could be attributed to supraphysiological levels of steroids (such as estradiol and other steroid hormones + ZEN and its metabolites) [49,50]. However, dietary ZEN can exert multiple effects on cells with estrogen receptors in the following ways: (i) as a factor responsible for an increase in the levels of estrogen hormones (not only estradiol) due to the fact that ZEN is captured by intestinal estrogen receptors, which induces qualitative changes in these estrogen receptors and triggers their expression, in particular estrogen receptor beta expression in the descending colon [31]; (ii) the obtained results seem to confirm that ZEN is not a substrate [38] for the cytochrome P450 1A1 enzyme in gilts before puberty; (iii) in gilts before puberty, the expression of CYP1A1 is silenced under the influence of ZEN, and it can limit uncontrolled proliferation by preventing conjugated enzymes from becoming detached and by increasing estrogen metabolism. In the first three weeks of exposure, no differences in *CYP1A1 m*RNA expression in the ascending colon were observed between group C and group E (Figure 2A). The above could be attributed to the polymorphism of cytochrome P450 1A1, or the fact that CYP1A1 alleles encoding enzymes with a higher metabolic activity [51,52] are not mapped near enzyme active sites. However, significant differences (*p* ≤ 0.01 and *p* ≤ 0.05) in the descending colon suggest that the administered ZEN dose exerted inhibitory effects in pre-pubertal gilts (Figure 2B). The presence of significant differences (*p* ≤ 0.01), particularly in the ascending colon (Figure 2A), in the remaining weeks of exposure (4–6), confirms that ZEN slows down gene expression. Kaci et al. [19] also found that ZEN strongly inhibits *CYP1A1* mRNA expression. Zhao et al. [18] observed that CYP enzymes are more often suppressed than induced. In turn, Mahato et al. [38] have argued that ZEN can act as both a substrate and an inhibitor of *CYP1A1 m*RNA expression.

In group C and group E, the mean values of *CYP1A1 m*RNA expression during the entire experiment were determined at 0.99 and 0.73, respectively, in the ascending colon (Figure 2A) and 0.57 and 0.43, respectively, in the descending colon (Figure 2B). In both groups, the lowest values were noted in the descending colon. The suppression of the examined gene suggests that the CYP1A1 enzyme was less involved in phase I of ZEN biotransformation in both parts of the colon in pre-pubertal gilts. It is possible that ZEN was bound to estrogen receptors to compensate for the physiological deficit of estradiol. The decrease in *CYP1A1 m*RNA expression could also be attributed to a protective mechanism that eliminates ZEN metabolites, in particular α-ZEL which is characterized by higher biological activity than the parent compound.

The suppression of *CYP1A1 m*RNA expression in the ascending colon in the final weeks of exposure (Figure 2A) and in the descending colon in all weeks of exposure (Figure 2B) [10,19] led to an increase in the concentration of ZEN (Figure 1) which is not a substrate for GSTπ1.

These results suggest that exposure to 40 μg ZEN/kg BW suppresses *CYP1A1* mRNA expression in pre-pubertal gilts in both segments of the large intestine (Figure 2). Zearalenone caused a significantly greater decrease in expression *CYP1A1* in the descending colon (0.43) than in the ascending colon (0.73).

#### 3.2.2. GSTπ1 Gene Encoding Metabolic Enzymes in Phase II of the Biotransformation Process

Microsomal GST enzymes participate in phase II of the biotransformation process [53]. It participates in the elimination of endogenous and exogenous toxins by binding them or facilitating their removal from cells. This enzyme protects the body against the harmful products of oxidative stress and prevents damage to nucleic acids and lipids [53,54]. It participates in the metabolism of steroid hormones and estrogen-like compounds, and it mediates the biosynthesis of leukotrienes and prostaglandins [54,55]. Glutathione S-transferase π1 not only participates in metabolite detoxification, but also binds ligands that initiate stress-induced cell apoptosis [56].

The results of the present study are difficult to interpret due to the lack of reference data. In group E, on most exposure dates, GSTπ1 gene expression was much more silenced in the ascending colon (Figure 3A) and slightly silenced in the descending colon (Figure 3B). In clinically healthy animals with subclinical ZEN mycotoxicosis, the minor overexpression of the GSTπ1 gene in the ascending colon indirectly proves that (i) animals are exposed to undesirable environmental contaminants such as ZEN, at the level of the colon; (ii) GSTπ1 activity decreased in cells with normal proliferative potential [57]; (iii) differences in expression in the analyzed segments of the colon suggest that exposure to an undesirable substance such as ZEN decreases the expression of the GSTπ1 gene in the ascending colon (Figure 3A) and decreases ZEN detoxification in the descending colon (Figure 3B), probably because this xenobiotic is relatively effectively detoxified in the ascending colon (Figure 3A) [58]; (iv) the observed differences in GSTπ1 gene expression levels (Figure 3B) suggest that this enzyme could be a new biomedical marker of subclinical ZEN mycotoxicosis in gilts before puberty, but further research is needed to validate this claim.

In group E, the expression of the *GSTπ1* gene was significantly lower (*p* ≤ 0.01) in the ascending colon in all weeks of exposure (Figure 3A). Only a minor decrease in the analyzed parameter was noted in the descending colon (Figure 3B). During the entire experiment, the presence of ZEN in the intestinal contents decreased *GSTπ1* expression to 0.39 in the ascending colon (0.96 in group C) (Figure 3A) and to 0.94 in the descending colon (1.07 in group C) (Figure 3B) on average. The above indicates that proliferation processes in the descending colon were similar in both groups (Figure 3B) [58]. It could be hypothesized that the slowing down of biotransformation processes in the descending colon decreased the concentration of ZEN (Figure 1) conjugated with reactive polar groups that were transformed into more water-soluble forms. As a result, substrate levels were too low to stimulate the expression of the *GSTπ1* gene.

The results noted in the descending colon (Figure 3B) were similar to those reported by Hokaiwado et al. [59]. The cited authors argued that *GSTπ1* gene suppression decreases cell proliferation, but it also weakens the enzyme’s protective and detoxifying effects [25]. Transcription factors that trigger cellular repair mechanisms are activated first. However, if genetic material is damaged, apoptosis is induced in cells, which can lead to controlled proliferation [57]. According to other researchers, the *GSTπ1* gene can be suppressed in response to chemical stress [60], such as exposure to ZEN. In enterocytes, *GSTπ1* expression was somewhat higher in group E (in weeks 1 and 3, not significant; Figure 3B) than in group C, possibly because the GSTπ1 enzyme was more involved in the process of GSH conjugation to ZEN [25].

#### 3.2.3. Summary

The expression of the *CYP1A1* and *GSTπ1* genes differed in two phases of the biotransformation process. In phase I, *CYP1A1 m*RNA expression in the ascending colon was suppressed in the last three weeks of exposure, which led to a substantial increase in ZEN concentration in the descending colon of pre-pubertal gilts. The resulting chemical stress strongly suppressed *GSTπ1* expression in the ascending colon.

In phase II, the concentration of ZEN in the descending colon increased (Figure 1) as a result of *CYP1A1* suppression in the ascending colon (Figure 2). Due to high ZEN levels in the descending colon (Figure 1), *CYP1A1 m*RNA expression was still more strongly suppressed in group E (Figure 3A), which undermined the effectiveness of phase I of the enzymatic biotransformation process. The above decreased the availability of the substrate (ZEN conjugated with reactive polar groups) for initiating phase II of the detoxification processes, which could have contributed to the increased accumulation of ZEN in the mucosa of the descending colon (Figure 1) in the last three weeks of exposure (weeks 4, 5, and 6).

The results of this study indicate that exposure to a dose of 40 µg ZEN/kg BW for 42 days disrupted the expression of the *CYP1A1* and *GSTπ1* genes that participate in the enzymatic biotransformation of ZEN in both analyzed segments of the large intestine in pre-pubertal gilts.

## 4. Materials and Methods

This investigation was performed at the Department of Veterinary Prevention and Feed Hygiene, Faculty of Veterinary Medicine of the University of Warmia and Mazury in Olsztyn, Poland, on 36 clinically healthy young gilts with an initial BW of 25 ± 2 kg at the age of 75 ± 5 days. The gilts were penned in groups with ad libitum access to water.

### 4.1. Experimental Design

Animals were randomly divided into an experimental group (E = ZEN; n = 18) and a control group (C, n = 18), which is in line with the suggestions from Smith et al. [61], Heberer et al. [62], and Directive 2010/63/EU [63]. Group E gilts were given ZEN orally at a dose of 40 μg ZEN/kg BW (Table 1). At the time of designing the experiment, the above value was equivalent to the no-observed-adverse-effect level (NOAEL) stipulated in the EFSA guidelines [50], which is 10 times higher than the current dose [21,64]. Animals from group C received a placebo. The experiment lasted 42 days. The ZEN dose was adjusted to the BW of group E animals. Zearalenone was administered in enteric-coated capsules to avoid problems with uneven food intake. On each day of the experiment, the mycotoxin was administered orally before the morning feeding. Feed was used as a vector. Group C gilts received a placebo, i.e., the same gastro-resistant capsules with the vector, but without ZEN. Zearalenone was dissolved in 500 μL of 96% C_2_H_5_OH (96% ethyl, SWW 2442-90, Polskie Odczynniki Chemiczne SA, Gliwice, Poland) to obtain the dose needed (adjusted to BW). The obtained solutions were stored at a temperature of 20 °C for 12 h. Pre-pubertal gilts were weighed once a week to adjust ZEN doses to the needs of each animal. Three gilts from each experiment group were killed on day 7 (1st week of exposure), 14 (2nd week of exposure), 21 (3rd week of exposure), 28 (4th week of exposure), 35 (5th week of exposure), and 42 (6th week of exposure). On each of the above dates, the animals were euthanized by intravenous administration of sodium pentobarbital (Fatro, Ozzano Emilia, BO, Italy). Tissue samples (approx. 1.0 cm × 1.5 cm) from the middle parts of both examined colon sections were collected immediately after cardiac arrest. Samples were washed with phosphate buffer and prepared for toxicological analyses and gene expression assessment. Laboratory analyses were conducted within one month after tissue sampling.

### 4.2. Experimental Feed

During the experiment, gilts received pelleted feed twice a day (8 a.m. and 5 p.m.). The chemical composition of the diets given to group C and group E gilts was determined with a NIRS-DS2500 F analyzer (FOSS, Hillerød, Denmark) with a scanning range of 850–2500 nm (Table 2). Pre-pubertal gilts were kept in pens with unlimited access to water.

### 4.3. Toxicological Studies

#### 4.3.1. Mycotoxin Analysis in Feed

The feed was analyzed for the presence of ZEN and DON, and the concentration of mycotoxins was determined by separation on immunoafinitive columns (Zearala-TestTM Zearalenone Testing System, G1012, VICAM, Watertown, MA, USA; DON-TestTM DON Testing System, VICAM, Watertown, MA, USA), high-performance liquid chromatography (Agilent 1260 HPLC system, Santa Clara, CA, USA), and mass spectrometry (MS, Agilent 6470) with the use of chromatographic columns (Atlantis T3, 3 μm, 3 mm × 150 mm, column No. 186003723, Waters, AN Etten-Leur, Ireland). The mycotoxins were separated by a mobile phase consisting of acetonitrile, water, and methanol (46:46:8, *v/v/v*) with a flow coefficient of 0.4 mL/min. The limit of quantification (LOQ) was set at 2 ng/g for ZEN and 5 ng/g for DON. Zearalenone and its metabolites were quantified at the Department of Veterinary Prevention and Feed Hygiene, Faculty of Veterinary Medicine of the University of Warmia and Mazury in Olsztyn, Poland [65].

#### 4.3.2. Biotransformation of ZEN

Zearalenone was synthesized and standardized by Prof. Piotr Goliński’s team at the laboratory of the Department of Chemistry of the Poznań University of Life Sciences, on the basis of a previously developed procedure [66]. The conditions and efficiency of ZEN formation and the method of its purification to the degree of crystalline standard purity were developed. The purity of the compound was determined by controlling its melting point, high-performance liquid chromatography (elution profile with retention times), spectroscopic instrumental analysis, and a comparison of the obtained results with a computer database—a purity value of 99.8% was obtained. The average efficiency of ZEN biosynthesis for the toxin-causing isolates used was 120 mg/kg, while the concentration of the toxin in the feeds ranged from 15.58 to 95.30 ng/g.

##### Extraction and Purification

Zearalenone, α-ZEL, and β-ZEL were extracted from tissue samples using immunoaffinity columns (Zearala-TestTM Zearalenone Testing System, G1012, VICAM, Watertown, MA, USA) according to the manufacturer’s instructions. The eluates were placed in a water bath at 50 °C and evaporated in nitrogen stream. Until chromatographic analysis, the dry residues were stored at −20 °C. The results were verified by mass spectrometry and the procedure was monitored using internal standards.

##### Chromatographic Quantification of ZEN, α-ZEL, and β-ZEL

ZEN, α-ZEL, and β-ZEL tissue concentrations were determined using an Agilent 1260 liquid chromatograph (LC) and an Agilent 6470 mass spectrometer (MS). The prepared samples were analyzed using an Agilent ZORBAX high-resolution HPLC column (Agilent Eclipse Plus C18; 2.1 × 50 mm, 1.8 µm) in a gradient elution program. The mobile phase consisted of 0.1% (*v*/*v*) formic acid dissolved in water (solvent A) and 0.1% (*v*/*v*) formic acid dissolved in acetonitrile (solvent B). The gradient program began with 20% B, which was increased to 100% B after 4.0 min and decreased to 20% B after 0.1 min.

##### Gradient Elution Conditions

Mycotoxins were quantified using external standards and were expressed in ppb (ng/mL). Calibration standards are fitted to the detector to eliminate detector influences that may reduce test sensitivity. Following the procedure described for the remaining samples, calibration standards were dissolved in the matrix samples (matrix-matched calibration). The material for the preparation of calibration standards was free from mycotoxins. The signal-to-noise ratio (3:1) was determined based on the limit of detection (LOD) for ZEN, α-ZEL, and β-ZEL. The LOQ was defined as three times the LOD.

The specificity of the method was assessed by comparing the chromatograms of empty samples with the chromatograms of enriched tissues samples.

##### Mass Spectrometry Condition

Electrospray ionization (ESI) mass spectrometry was performed in negative ion mode. MS/MS indices were optimized for all compounds. Using a six-level calibration curve, linearity was assessed. The optimized conditions for the mycotoxins analyzed are shown in Table 3.

##### Statistical Analysis

The results were statistically processed at the Department of Discrete Mathematics and Theoretical Computer Science, Faculty of Mathematics and Computer Science of the University of Warmia and Mazury in Olsztyn. At different sampling dates, the bioavailability of ZEN and its metabolites was determined in the intestinal tissues of pre-pubertal gilts in the experimental and control groups. The results were presented as means (±) and standard deviation (SD) values. The following indices were calculated as follows: (i) differences between the mean values within the colonic tissue examined in each week of exposure—in animals receiving the same doses of ZEN; (ii) differences between the mean values in the ascending and descending colon over the six weeks of exposure. In both tests, the differences between the means were determined by the unidirectional analysis of variance (ANOVA) method. If the differences between the groups were statistically significant, the Tukey Honestly Significant Difference test (HSD) identified significantly different pairs. If all values in either group were lower than the LOD (mean and variance were equal to 0), the values in the other group were processed using a unidirectional analysis of variance, and the differences between the means in that group were compared with the zero difference between the population means in Student’s *t*-test. The differences between groups were assessed using Student’s *t*-test. The results of each analysis were considered highly significant at *p* < 0.01 (**) and significant at 0.01 < *p* < 0.05 (*). Data were processed in the Statistica v. 13 program (TIBCO Software Inc., San Ramon, CA, USA, 2017).

### 4.4. Expression of CYP1A1 and GSTπ1 [65]

#### 4.4.1. Sampling for RNA Extraction

Colon tissue samples were collected immediately after cardiac arrest. According to the manufacturer’s instructions (Sigma-Aldrich, Taufkirchen, Germany), samples were stored in RNAlater solution.

#### 4.4.2. RNA Extraction and cDNA Synthesis

According to the manufacturer’s protocol, total RNA was extracted from tissues preserved in RNAlater (ca. 20 mg per sample; n 1/43 in each group) using the Total RNA Mini kit (A&A Biotechnology, Gdynia, Poland). RNA samples were incubated in ribonuclease-free DNAse I (Roche Diagnostics, Mannheim, Germany) to prevent contamination with genomic DNA. Overall RNA quality and purity from all samples were assessed using a BioPhotometer (Eppendorf, Germany) and the results were used for cDNA synthesis using the RevertAid™ First Strand cDNA Synthesis Kit (Fermentas, Burlington, ON, Canada). According to the manufacturer’s protocol, the reaction was performed using a cDNA synthesis reaction mixture for each sample, which consisted of 1 µg of total RNA and 0.5 µg of oligo (dT)18 primers. The first synthesized cDNA chain was stored at −20 °C pending further analysis.

#### 4.4.3. qPCR

Real-time PCR primers for target mRNA were designed using the Primer-BLAST tool [67] based on reference species (Table 4). Real-time PCR was performed on an ABI 7500 Real-Time PCR system (Applied Biosystems, Foster City, CA, USA) in single plex mode; further procedures were performed according to the manufacturer’s instructions. Each PCR reaction tube contained 10 μL of the FastStart SYBR Green Master ROX mix (Roche Diagnostics), 0.25–0.5 μM of each primer (forward and reverse; Table 4), and 1 μL of the previously synthesized cDNA as a template, supplemented with PCR-grade H_2_O to a final volume of 20 μL. The reaction was performed under the following standard thermal conditions: 95 °C for 10 min, followed by 45 cycles of 95 °C for 15 s and 60 °C for 1 min. Negative water controls (NTCs) were included to rule out cross-contamination. The quality of PCR products was verified by melting curve analysis, followed by agarose gel electrophoresis. The specificity of the designed primer pairs was confirmed by sequencing the obtained PCR products (RevertAid™ First Strand cDNA Synthesis Kit, Fermentas).

For qPCR, the quantitative cycle (Cq) values were converted to copy numbers using a standard curve (Cq versus logarithm of copy number) according to the methodology proposed by Arukwe [69] and presented by Spachmo and Arukwe [70].

The use of the standard curve was based on the assumption that the unknown samples had the same enhancement efficiency (typically above 90%), and this assumption was verified before extrapolating the unknown standards onto the standard curve. To generate the standard curves, the purified PCR products of each mRNA were used to prepare a series of six 10-fold dilutions with a known copy number. These dilutions were used as templates in RT-PCR. Cq values for each dilution series were calculated relative to the logarithm of the copy number and were used to extrapolate unknown samples to the copy numbers. The mRNA copy numbers in samples collected from both groups during the experiment were divided by the mean copy numbers in group C determined on day 0 of the experiment to calculate relative expression values as expression ratios (R).

#### 4.4.4. Statistical Analysis

Mean (±) and SD values of *CYP1A1* and *GSTπ1* gene expression in the ascending and descending colon were presented for each sample. The results were analyzed in Statistica software (StatSoft Inc., Tulsa, OK, USA). Mean values in groups C and E were compared using repeated measures ANOVA based on the dose of ZEN administered to young gilts. If ANOVA revealed differences between groups, Tukey’s HSD test was used to determine which pairs of group means were significantly different from each other. The ANOVA assumes that data in each group are normally distributed and that variance in each group is equal. Therefore, group samples were collected from a population characterized by a normal distribution and homogeneity of variance. If the above assumptions were not met, the equality of means was analyzed using the Kruskal–Wallis test, a non-parametric alternative to ANOVA, and multiple comparisons were performed. The results of the non-parametric test were considered if differences were found between groups.

## Figures and Tables

**Table 1:** Daily feed intake in a restricted feeding regimen (kg/day), average ZEN concentration per kg of feed [65].

Week of Exposure	Feed Intake	Total ZEN Dose	
	kg/Day	µg/Gilt	µg/kg Feed
1	1.1	280	1014
2	1.0	560	972
3	1.3	840	1014
4	1.6	1120	987
5	1.9	1400	995
6	1.7	1680	957

**Table 2:** Diet composition for pre-pubertal gilts (1st stage of rearing).

Percentage Content of Feed Ingredients		Nutritional Value of Diets	
Barley	27.65	Metabolizable energy MJ/kg	12.575
Wheat	17.5	Total protein (%)	16.8
Triticale	15.0	Digestible protein (%)	13.95
Maize	17.5	Lysine (g/kg)	9.975
Soybean meal, 46%	16.0	Methionine + Cysteine (g/kg)	6.25
Rapeseed meal	3.5	Calcium (g/kg)	8.05
Limestone	0.35	Total phosphorus (g/kg)	5.75
Premix ^1^	2.5	Available phosphorus (g/kg)	3.1
		Sodium (g/kg)	1.5

Abbreviations: ^1^ Vitamin and mineral premix composition per kg: vitamin A—500.000 IU; Fe—5000 mg; vitamin D_3_—100.000 IU; Zn—5000 mg; vitamin E (alpha-tocopherol)—2000 mg; Mn—3000 mg; vitamin K—150 mg; copper (CuSO_4_·5H_2_O)—500 mg; vitamin B_1_—100 mg; Co—20 mg; vitamin B_2_—300 mg; iodine—40 mg; vitamin B_6_—150 mg; Se—15 mg; vitamin B_12_—1500 μg; niacin—1200 mg; pantothenic acid—600 mg; L-threonine—2.3 g; folic acid—50 mg; tryptophan—1.1 g; biotin—7500 μg; phytase + choline—10 g; ToyoCerin probiotic + calcium—250 g; magnesium—5 g.

**Table 3:** Optimized conditions for mycotoxins to be tested.

Analyte	Precursor	Quantification Ion	Confirmation Ion	LOD (ng/mL)	LOQ (ng/mL)	Linearity (%R^2^)
** ZEN **	317.1	273.3	187.1	0.03	0.1	0.999
** α-ZEL **	319.2	275.2	160.1	0.3	0.9	0.997
** β-ZEL **	319.2	275.2	160.1	0.3	1	0.993

**Table 4:** Real-time PCR primers used in the study [52].

Primer	Sequence (5’→3’)		Amplicon Length (bp)	References
**CYP1A1**	Forward	cagagccgcagcagccaccttg	226	[65]
	Reverse	ggctcttgcccaaggtcagcac		
**GST** **π** **1**	Forward	acctgcttcggattcaccag	178	[65]
	Reverse	ctccagccacaaagccctta		
**β-actin**	Forward	catcaccatcggcaaaga	237	[68]
	Reverse	Gcgtagaggtccttcctgatgt		

## Data Availability

The original contributions presented in this study are included in the article. Further inquiries can be directed to the corresponding author.
